# Perceived Motivational Climate and Stages of Exercise Behavior Change: Mediating Roles of Motivation Within and Beyond Physical Education Class

**DOI:** 10.3389/fpsyg.2021.737461

**Published:** 2021-10-25

**Authors:** Xiaojing Wu, Xiaosong Gai, Tianfeng Yu, Haifeng Yu, Yu Zhang

**Affiliations:** ^1^School of Psychology, Northeast Normal University, Changchun, China; ^2^Hong Qi Senior High of Dalian City, Dalian, China; ^3^Haikou Middle School, Haikou, China

**Keywords:** motivational climate, physical education, physical activity, achievement goal theory, autonomous motivation

## Abstract

This study examined the relationship between the perceived motivational climate in physical education (PE) classes and the stage of exercise behavior change among 322 high school students in northeastern China. Furthermore, the chain-mediating effects of autonomous motivation in PE and the five types of motivation (interest, competence, social relatedness, appearance, and health) in leisure-time physical activity (PA) were examined. Results showed that autonomous motivation in PE and the five types of motivation in leisure-time PA had chain-mediating effects on the relationships between the perceived mastery- and performance-oriented motivational climate and the stage of exercise behavior change in the whole sample (controlling for gender). Specifically, a perceived mastery-oriented motivational climate promoted autonomous motivation in PE, which, in turn, increased the five types of motivation in leisure-time PA and led to a higher stage of exercise behavior change. Conversely, a perceived performance-oriented motivational climate reduced autonomous motivation in PE which, in turn, decreased the five types of motivation in leisure-time PA and led to earlier stages of exercise behavior change. The patterns of the chain-mediating effects of autonomous motivation in PE and the five types of motivation in leisure-time PA were somewhat similar in girls and boys. The results suggested that PE teachers should create mastery-oriented climates and avoid performance-oriented motivational climates, which may promote intrinsic or identified motivations of students for PA within and beyond PE classes, thereby affecting the leisure-time PA of students.

## Introduction

Physical activity can accelerate the physical development of adolescents (Smith et al., 2008; Guan and Zhu, 2013a,b) and cultivate character strengths such as mental toughness (Jones and Parker, 2013), confidence, and compassion (Linver et al., 2009). However, ~84.3% of adolescents (aged 11–17 years) in China did not meet the recommended PA levels [≥60 min of moderate-to-vigorous intensity physical activity (PA) daily; Guthold et al., 2020]. This suggests the need for effective ways to encourage Chinese adolescents to engage in PA regularly (Pitkethly et al., 2019). Given that schools and physical education (PE) teachers have access to almost all adolescents, it has been proposed that schools and PE are effective ways to affect the health of people (Sallis et al., 2012) and increase the PA of adolescents (Guthold et al., 2020).

### The Motivational Climate in PE and Leisure-Time PA

The perceptions of students on motivational climates created by PE teachers may affect their actual leisure-time PA behavior. Based on the achievement goal theory (AGT), Ames (1992) proposed that mastery- and performance-oriented motivational climates can be used to reflect the perceptions of students on class. A mastery-oriented motivational climate emphasizes the efforts and cooperation of participants, reinforces personal improvement, stresses the fundamental role of each member in the group, and views mistakes as part of the learning process. Conversely, a performance-oriented motivational climate encourages intra-team member rivalry, unequal recognition, and penalties for mistakes (Newton et al., 2000). The AGT postulates that perceptions of a mastery-oriented motivational climate result in more positive consequences (e.g., health and fitness outcomes and skill-based outcomes) as compared with perceptions of a performance-oriented motivational climate (Duda and Balaguer, 2007). Consistent with the AGT, previous studies found that a perceived mastery-oriented motivational climate positively predicted many leisure-time PA indicators, such as the number of days within a week that participants engaged in moderate-to-vigorous PA for at least 60 min (Jaakkola et al., 2017), the frequency of vigorous PA (Theodosiou and Papaioannou, 2006) and low-intensity PA (Grastén, 2016), and whether or not participants engaged in PA at all (Ommundsen and Kvalø, 2007). In contrast, a perceived performance-oriented motivational climate has been found to have no significant effect on the time that adolescents participate in leisure-time PA (Ommundsen and Kvalø, 2007; Jaakkola et al., 2017).

The stage of exercise behavior change is one of the PA indicators. It reflects the stage of motivational readiness of an individual for exercise and their current participation in regular exercise (regular exercise refers to exercising three or more times a week, for 20 min or more each time; Marcus et al., 1992). According to the Trans-Theoretical Model (TTM), the stages of exercise behavior change are differentiated into five stages: pre-contemplation—inactive, with no intention to start exercising; contemplation—inactive, with the intention to start regular exercise within the next 6 months; preparation—participating in exercise irregularly; action—participating in regular exercise for <6 months; maintenance—participating in regular exercise for 6 or more months (Marcus et al., 1992). It was previously reported that students who were physically active had higher physical fitness, wellbeing, health satisfaction, subjective weight, and body satisfaction than those who were physically inactive (Duan et al., 2012; Rostami et al., 2017). On the other hand, those who engaged in regular exercise exhibited lower levels of state anger and higher levels of anger control than those who did not (Kim et al., 2019). However, it has not been directly examined whether a perceived motivational climate could predict the stage of exercise behavior change. This study attempts to fill this gap. Previous studies have found that the perceptions students have of a mastery-oriented motivational climate positively predict their mastery goals (Barkoukis et al., 2007; Smith et al., 2009), and students with higher levels of mastery goals were more likely in the active stages than the inactive stages of behavior change (Vílchez and Ruiz, 2016). Based on these findings, a perceived motivational climate may predict the stage of exercise behavior change. In this study, we will examine whether a perceived motivational climate in PE predicts the stage of exercise behavior change (along with other PA indicators: length of time participating in low- and high-intensity PA, PA types) in senior high school students.

### The Pathways Through Which Motivational Climates in PE Translate Into Leisure-Time PA

The examination of the pathways through which perceived motivational climates in PE translates into leisure-time PA is well warranted (Jaakkola et al., 2017), as this could help us not only understand the underlying mechanisms, but also provide ways to promote the engagement of adolescents in leisure-time PA.

The existing literature offers insights into how the perceived motivational climate in PE classes may translate into leisure-time PA. It has been found that the perceived mastery-oriented motivational climate of PE classes has a positive effect on the autonomous motivation of students in PE (Ommundsen and Kvalø, 2007; Barkoukis and Hagger, 2013), while the perceived performance-oriented motivational climate of PE classes has a neutral or negative effect on autonomous motivation in PE (Standage et al., 2003; Ommundsen and Kvalø, 2007; Barkoukis and Hagger, 2013; Cid et al., 2019). According to Vallerand's (2007) model, individuals store motivational experiences in different life contexts as motivational schemas. When the individual encounters similar or related contexts, these schemas can serve to promote or inhibit motivation toward engaging in this context. Therefore, the motivational experience in PE could translate into motivations for leisure-time PA (Wang and Chen, 2020). Furthermore, the trans-contextual model of motivation (TCM) proposes that perceived autonomy support in PE promotes autonomous motivation in PE, which, in turn, increases autonomous motivation in leisure-time PA and leads to more (intended and actual) leisure-time PA behavior (Hagger et al., 2003). Barkoukis and Hagger (2013) extended the TCM by integrating the perceptions of a motivational climate in PE as predictors of autonomous motivation in PE, and they found that a perceived mastery-oriented motivational climate translated into leisure-time PA frequency through a chain-mediating effect of autonomous motivation in PE, autonomous motivation in leisure-time PA, subjective norms, and leisure-time PA intention. Wang and Chen (2020) found that autonomous motivation in PE had an indirect effect on out-of-school PA through autonomous motivation in leisure-time PA. Therefore, a motivational climate could translate into leisure-time PA through a chain-mediating effect of autonomous motivation in PE and autonomous motivation in leisure-time PA.

However, the chain-mediating effects of autonomous motivation in PE and the different types of motivation in leisure-time PA on the relationships between perceived motivational climates and leisure-time PA have not been examined. Frederick and Ryan (1993) found that there were five types of motivation that describe the specific reasons or goals for leisure-time PA: interest, competence, appearance, health, and social relatedness. Researchers regard interest, competence, and social relatedness as relatively intrinsic motivations, whereas health and appearance are considered relatively extrinsic motivations (Goguen Carpenter et al., 2017; Beauchemin et al., 2019). One study suggested that motivations that focused on enjoyment, competence, and social interactions were associated with greater exercise adherence than fitness and appearance motivations (Ryan et al., 1997). However, other studies have found that both intrinsic and extrinsic motivations positively predict leisure-time PA. Some examples include: Frederick and Ryan (1993) found that body-related motivations positively predict days per week of exercise, Goguen Carpenter et al. (2017) found that interest, competence, and health motivations positively predict participation in group-based PA, and Beauchemin et al. (2019) found that all five types of leisure-time PA motivations are positively related to walking frequency and distance. Compared to pre-action stages, higher relatively extrinsic (health and appearance) and relatively intrinsic (interest, competence, and social relatedness) motivations were both observed in the action and maintenance stages (Vancampfort et al., 2017). Considering the nature of appearance and health motivations (i.e., those in which individuals identify the values and effects of PA in improving their appearance and health), we speculated that these two motivations were essentially identified motivations that positively predict the leisure-time PA behavior of an individual (Hutmacher et al., 2020). Consistent with this speculation, one study found that both health motivation and appearance motivation in leisure-time PA are positively related to identified motivation in leisure-time PA (Sibley et al., 2013). A cross-sectional study found that perceived mastery-oriented motivational climate in PE displays a positive effect on interest and health as reasons to exercise and a neutral effect on attractiveness and fitness as reasons to exercise. In addition, perceived performance-oriented motivational climate has a neutral effect on these four reasons to exercise (Brown and Fry, 2013). However, the study did not examine whether the perceived motivational climate in PE classes correlated with all five types of motivation in leisure-time PA (Brown and Fry, 2013). To our knowledge, no studies have examined whether perceived motivational climates could translate into the stage of exercise behavior change (or any other PA indicators) through chain-mediating effects of autonomous motivation in PE class and the five types of motivation in leisure-time PA. This study attempts to fill these gaps in the research.

### The Present Study

In summary, our main purpose was three-fold. First, we examined whether the perceived motivational climates in PE predict the stage of exercise behavior change (along with other PA indicators: length of time participating in low- and high-intensity PA and PA types). Second, we examined whether the perceived motivational climates in PE predict the five types of motivation in leisure-time PA. Third, we examined the chain-mediating effects of autonomous motivation in PE class and the five types of motivation in leisure-time PA on the relationships between perceived mastery- and performance-oriented motivational climates and the stage of exercise behavior change. To corroborate the chain-mediating effects, length of time participating in low- and high-intensity PA and PA types were also included in the path analysis. To answer these questions, we conducted this study with senior high school students.

We proposed the following hypotheses:

**Hypothesis 1**. A perceived mastery-oriented motivational climate in PE would have a positive effect on the stage of exercise behavior change (and other PA indicators), and a perceived performance-oriented motivational climate would have a negative or neutral effect on the stage of exercise behavior change (and other PA indicators).
**Hypothesis 2**. A perceived mastery-oriented motivational climate in PE would increase the relatively intrinsic motivations (interest, competence, and social relatedness) of students in leisure-time PA. A perceived performance-oriented motivational climate would decrease the relatively intrinsic motivations of students in leisure-time PA (Brown and Fry, 2013). The relationships between perceived motivational climates and relatively extrinsic motivations (health and appearance) were more exploratory.
**Hypothesis 3**. According to the extended TCM (Barkoukis and Hagger, 2013), autonomous motivation in PE class and relatively intrinsic motivations in leisure-time PA were expected to have chain-mediating effects on the relationships between a perceived motivational climate and the stage of exercise behavior change (and other PA indicators). Specifically, a perceived mastery-oriented motivational climate was expected to have a positive effect on autonomous motivation in PE (Hypothesis 3a) (Barkoukis and Hagger, 2013). As previous studies have found that a perceived performance-oriented motivational climate was negatively or not associated with autonomous motivation within PE (Standage et al., 2003; Ommundsen and Kvalø, 2007; Barkoukis and Hagger, 2013), its predictive effect on autonomous motivation in PE was more exploratory. Autonomous motivation in PE was expected to have a positive effect on relatively intrinsic motivations in leisure-time PA (Hypothesis 3b). Finally, relatively intrinsic motivations in leisure-time PA were expected to have positive effects on the stage of exercise behavior change (and other PA indicators; Hypothesis 3c). The chain-mediating effects of autonomous motivation in PE and relatively extrinsic motivations in leisure-time PA on the relationships between motivational climates and PA indicators were more exploratory.

In addition, one previous study found that Chinese junior high secondary school girls participate in less leisure-time PA than boys (Si et al., 2015) and have higher levels of perceptions of a performance-oriented motivational climate in class (Jiang and Liu, 2006). We explored whether there were gender differences in perceived motivational climates, autonomous motivation in PE, motivations in leisure-time PA, and leisure-time PA indicators. Lastly, we examined the chain-mediating effects of motivations within and beyond PE in the whole sample (controlling for gender) and in the girls and boys, respectively.

## Method

### Participants

Adolescents (*N* = 344; 138 boys, 206 girls; *n* = 124 in 10th grade, *n* = 220 in 11th grade) from nine PE classes of a high school in northeast China were recruited for this study. Twenty-two students were excluded because of missing data on two PA indicators (length of time participating in low- or high-intensity PA). Therefore, the final sample consisted of 322 students (121 boys, 201 girls; *n* = 111 in 10th grade, *n* = 211 in 11th grade). The mean age of participants was 16.76 years (*SD* = 0.68, range = 14.73–19.21 years).

### Measures

#### Motivational Climate

The Motivational Climate Scale for Youth Sports was developed by Smith et al. (2008) and revised for Chinese adolescents by Zhang (2016). This scale consists of two subscales measuring mastery- and performance-oriented motivational climates, with each subscale having six items (Smith et al., 2008). For the performance-oriented dimension, Zhang (2016) found that the discriminant validity of one item was lower than 0.4 among Chinese adolescents. Therefore, this item was not included in our measures. Responses were indicated on a 5-point Likert scale from 1 = *strongly disagree* to 5 = *strongly agree*. In this study, the Cronbach's alpha coefficients for the mastery- and performance-oriented dimensions were 0.89 and 0.71, respectively. A confirmatory factor analysis (CFA) demonstrated satisfactory parameters [χ^2^ = 133.85; *df* = 43; RMSEA = 0.081 (90% CI = 0.066–0.097); CFI = 0.941; TLI = 0.924; SRMR = 0.067].

#### Motivational Regulations in PE

Motivational regulations in PE were assessed with the revised version of Perceived Locus of Causality Scale for PE (Vlachopoulos et al., 2011), which consisted of 19 items with *five* subscales comprising intrinsic motivation (4 items), identified regulation (4 items), introjected regulation (4 items), external regulation (3 items), and amotivation (4 items). Participants rated each item on a 7-point Likert scale from 1 (*does not correspond at all*) to 7 (*corresponds exactly*). In this study, the factor loading of one item measuring identified regulation was lower than 0.4. Therefore, it was removed from the analyses. Reliabilities were acceptable for intrinsic motivation (Cronbach's α = 0.8), identified regulation (Cronbach's α = 0.87), introjected regulation (Cronbach's α = 0.68), external regulation (Cronbach's α = 0.78), and amotivation (Cronbach's α = 0.73). The CFA confirmed the factor structure of the five motivations scales with the exception of the TLI, which was slightly lower than the recommended threshold [i.e., 0.9; χ^2^ = 367.97, *df* = 125, RMSEA = 0.078 (90% CI = 0.069–0.087), CFI = 0.912, TLI = 0.892, SRMR = 0.07]. Modification indices showed that there was a high error covariance between one item measuring external regulation (i.e., “Because in this way I will not get a low grade”) and other items measuring introjected regulation (i.e., “Because I would feel bad if the teacher thought that I am not good at PE”). Since these two items were both related to the performance of students in PE, we modified the measurement model by connecting the error terms of these two items. After this modification, the CFA demonstrated a satisfactory parameter [χ^2^ = 340.65; *df* = 124; RMSEA = 0.074 (90% CI = 0.064–0.083); CFI = 0.921; TLI = 0.903; SRMR = 0.068]. According to the suggestion made by Vlachopoulos et al. (2011), the subscale scores were combined into a composite autonomous motivation index (Relative Autonomy Index, RAI), computed using the following formula: RAI = 2 × (intrinsic motivation) + (identified regulation)–(introjected regulation)−2 × (external regulation)−3 × (amotivation). The higher the RAI, the higher the level of autonomous motivation.

#### Motivations in Leisure-Time PA

The Motivation for Leisure-time PA Scale was developed by Ryan et al. (1997) and revised for Chinese adolescents by Yu (2008). Each item was rated on a 7-point Likert scale ranging from 1 (does not correspond at all) to 7 (corresponds exactly). It consisted of 29 items with five subscales comprising interest motivation (5 items), appearance motivation (5 items), competence motivation (6 items), health motivation (6 items), and social relatedness motivation (7 items). In this study, the Cronbach's alpha coefficients were 0.91 for interest motivation, 0.89 for appearance motivation, 0.9 for competence motivation, 0.85 for health motivation, and 0.89 for social relatedness motivation.

#### Physical Activity

Leisure-time PA included four indicators in this study: PA stage, length of time participating in low-intensity PA, length of time participating in high-intensity PA, and PA types.

For the PA stage, the Physical Activity Stage of Change Questionnaire (Marcus et al., 1992) was used to assess the stage of exercise behavior change of adolescents. It consists of four items: “Usually, I am active in physical activity” (Item 1); “I currently do not exercise; however, I intend to exercise in 6 months” (Item 2); “I currently exercise regularly; however, I have only begun doing so within the past 6 months” (Item 3); “I currently exercise regularly and have been doing so for longer than 6 months” (Item 4). The response for each item was either *yes* (1) or *no* (0). It was explained to participants that regular exercise refers to exercising three or more times a week, each for 20 min or more. If the answers for Item 1 and Item 2 were both no, participants were in the “pre-contemplation stage.” If the answer for Item 1 was no and for Item 2 was yes, participants were in the “contemplation stage.” If the answer for Item 1 was yes, and for Item 3 and Item 4 were no, participants were in the “preparation stage.” If the answers for Item 1 and Item 3 were yes, and for Item 4 was no, participants were in the “action stage.” Finally, if the answers for Item 1 and Item 4 were yes, participants were in the “maintenance stage.” The Cronbach's alpha coefficient was 0.68 for the Physical Activity Stage of Change Questionnaire. The concurrent validity of the stage of exercise behavior change with strenuous and moderate exercise has been established (Schumann et al., 2002). In this study, the stage of exercise behavior change was positively related to the other three PA indicators (*r*s > 0.19, *p* < 0.01). Since these five stages show a gradual ascending trend in intention to exercise and participation in regular PA, the stage of exercise behavior change could be regarded as an ordinal variable. Therefore, we assigned 1, 2, 3, 4, and 5 to the pre-contemplation stage, contemplation stage, preparation stage, action stage, and maintenance stage, respectively. It was found that, as the number of categories of outcome variable increased (five or more), the normal linear regression can be an acceptable alternative method with a decrease in the root mean square error and the estimated standard errors of the mediation effect and an increase in the statistical power (Liu et al., 2013). Thus, we regarded the stage of exercise behavior change as a continuous variable in the path analysis.

For the length of time participating in low- and high-intensity leisure-time PA, participants were asked to report on two items: “How many hours did you spend on low-intensity leisure-time PA (e.g., walking and jogging) per month?” and “How many hours did you spend on high-intensity (e.g., challenging, competitive, confrontational) leisure-time PA per month?” Low- and high-intensity leisure-time PA were positively correlated with each other (*r* = 0.59, *p* < 0.01).

For PA types, adolescents reported (yes or no) their involvement in a range of physical activities over the past half-year, including ball sports, athletics, and other types of PA (e.g., hiking, climbing, and taekwondo). Physical activity types were positively related to the other three PA indicators (*r*s > 0.17, *p* < 0.01).

### Procedure

Informed consent was obtained from the participants and teachers. Headteachers were trained to administer the survey. Students completed the questionnaires on tablet computers at school. They were told that their participation was completely voluntary and confidential. The study was approved by the Ethical Committee for Scientific Research at the institution of the researchers.

### Statistical Analyses

The descriptive statistics and correlation coefficients were calculated using IBM SPSS Statistics, version 21. The confirmatory factor analysis and path analysis were conducted in Mplus 8.3. We ran bootstrap arithmetic (2,000 times) to conduct the path analysis. As the five types of motivation in leisure-time PA were moderately to highly correlated with each other in the whole sample and in the girls and boys, respectively, the error items of the five types of motivation were connected in the path analysis. In order to reduce the impact of multicollinearity, we followed the method of Marsh et al. (2004) and constrained the paths that examined the predictive effects of different types of motivation on the four PA indicators to be equal. Since the focus of this study was to examine the relationships between perceived motivational climate and leisure-time PA, we established the direct paths from perceived mastery- and performance-oriented motivational climates to the four PA indicators in the path analysis.

## Results

### Preliminary Analyses

Harman's one-factor analysis was conducted to assess the common method bias in this study (Podsakoff et al., 2003). We used all items of the variables to conduct the exploratory factor analysis. The unrotated factor solution extracted 12 distinct factors that accounted for 66.97% of the total variance, with the first factor explaining 33.43%. No single factor emerged and accounted for most of the variance. Therefore, a common method bias was not a major concern in this study.

Table 1 presents the correlation coefficients for all variables in the whole sample. Perceived mastery-oriented motivational climate was positively related to the RAI in PE, the five types of motivation in leisure-time PA, the stage of exercise behavior change, and low- and high-intensity leisure-time PA. A perceived performance-oriented motivational climate was negatively related to the RAI of students in PE, the four types of motivation in leisure-time PA (interest, competence, health, and social relatedness), and low-intensity leisure-time PA. These results supported the first two hypotheses. The RAI in PE was positively related to the five types of motivation in leisure-time PA and the leisure-time PA indicators (excluding PA types). The five types of motivation in leisure-time PA were positively related to the four leisure-time PA indicators, except that appearance motivation was unrelated to low-intensity leisure-time PA.

**Table 1:** Means, SDs, and correlation coefficients between study variables in the whole sample.

	**1**	**2**	**3**	**4**	**5**	**6**	**7**	**8**	**9**	**10**	**11**	**12**	**13**	**14**	**15**	**16**	**17**
1. MO	1																
2. PO	−0.413**	1															
3. Amotivation	−0.412**	0.433**	1														
4. Extrinsic	−0.201**	0.336**	0.588**	1													
5. Introjected	0.022	0.140*	0.268**	0.558**	1												
6. Identified	0.383**	−0.205**	−0.356**	−0.208**	0.136*	1											
7. Intrinsic	0.466**	−0.353**	−0.668**	−0.461**	−0.127*	0.633**	1										
8. RAI	0.415**	−0.432**	−0.862**	−0.814**	−0.473**	0.541**	0.818**	1									
9. Interest	0.481**	−0.242**	−0.512**	−0.359**	−0.126*	0.662**	0.749**	0.663**	1								
10. Competence	0.449**	−0.175**	−0.395**	−0.268**	−0.010	0.707**	0.655**	0.551**	0.872**	1							
11. Appearance	0.278**	−0.054	−0.246**	−0.067	0.032	0.357**	0.393**	0.284**	0.491**	0.572**	1						
12. Health	0.467**	−0.203**	−0.425**	−0.231**	0.020	0.645**	0.634**	0.527**	0.817**	0.822**	0.663**	1					
13. Social	0.572**	−0.231**	−0.419**	−0.264**	−0.030	0.590**	0.637**	0.535**	0.777**	0.788**	0.562**	0.782**	1				
14. Low PA	0.167**	−0.157**	−0.151**	−0.099	0.002	0.183**	0.174**	0.171**	0.163**	0.191**	0.083	0.168**	0.214**	1			
15. High PA	0.231**	−0.080	−0.186**	−0.105	0.029	0.251**	0.234**	0.210**	0.309**	0.307**	0.121*	0.294**	0.317**	0.593**	1		
16. PA Types	0.102	−0.026	−0.038	−0.019	0.065	0.135*	0.127*	0.069	0.176**	0.188**	0.122*	0.167**	0.165**	0.205**	0.179**	1	
17. PA Stage	0.273**	−0.038	−0.215**	−0.211**	−0.071	0.407**	0.420**	0.355**	0.570**	0.570**	0.303**	0.519**	0.522**	0.195**	0.340**	0.193**	1
18. Gender	0.219**	0.020	−0.066	−0.082	−0.047	0.219**	0.209**	0.160**	0.309**	0.248**	0.025	0.278**	0.265**	0.138*	0.393**	−0.017	0.316**
*M*	4.20	2.15	1.71	2.12	2.66	5.48	5.95	5.36	5.86	5.72	5.59	5.81	5.65	7.47	4.25	7.55	3.70
*SD*	0.72	0.69	0.95	1.27	1.42	1.49	1.07	7.76	1.09	1.18	1.33	1.01	1.17	9.14	7.13	5.31	1.30

*M, mean; SD, standard deviation; MO, mastery-oriented motivational climate; PO, performance-oriented motivational climate; External, external regulation; Introjected, introjected regulation; Identified, identified regulation; Intrinsic, intrinsic motivation; RAI, Relative Autonomy Index in PE; Interest, interest motivation; Competence, competence motivation; Appearance, appearance motivation; Health, health motivation; Social, social relatedness motivation; PA, physical activity; low PA, low-intensity PA; high PA, high-intensity PA. Gender: 0, girl; 1, boy*.

* *p < 0.05*,

** *p < 0.01*.

We explored whether there were gender differences in perceived motivational climates, the RAI in PE, leisure-time PA motivations, and leisure-time PA. As shown in Table 1, compared with girls, boys had higher levels of perception of the mastery-oriented motivational climate, the RAI in PE, and higher levels of interest, health, competence, and social relatedness motivations in leisure-time PA. Furthermore, boys participated more in low- and high-intensity leisure-time PA and maintained a higher stage of exercise behavior change.

### Chain-Mediating Effects of the RAI in PE and Motivations in Leisure-Time PA on the Relationships Between Perceived Motivational Climates and Physical Activity

In this study, the RAI in PE class and the relatively intrinsic motivations in leisure-time PA were expected to have chain-mediating effects on the relationships between perceived motivational climates and PA indicators (Hypothesis 3). Therefore, the possible chain-mediating effects of the RAI in PE and the five types of motivation in leisure-time PA on the relationships between perceived motivational climates and the stage of exercise behavior change with other PA indicators were examined using a path analysis in the whole sample (controlling for gender) and in girls and boys, respectively.

#### Chain-Mediating Effects of the RAI in PE and Motivations in Leisure-Time PA in the Whole Sample

**Figure 1:**
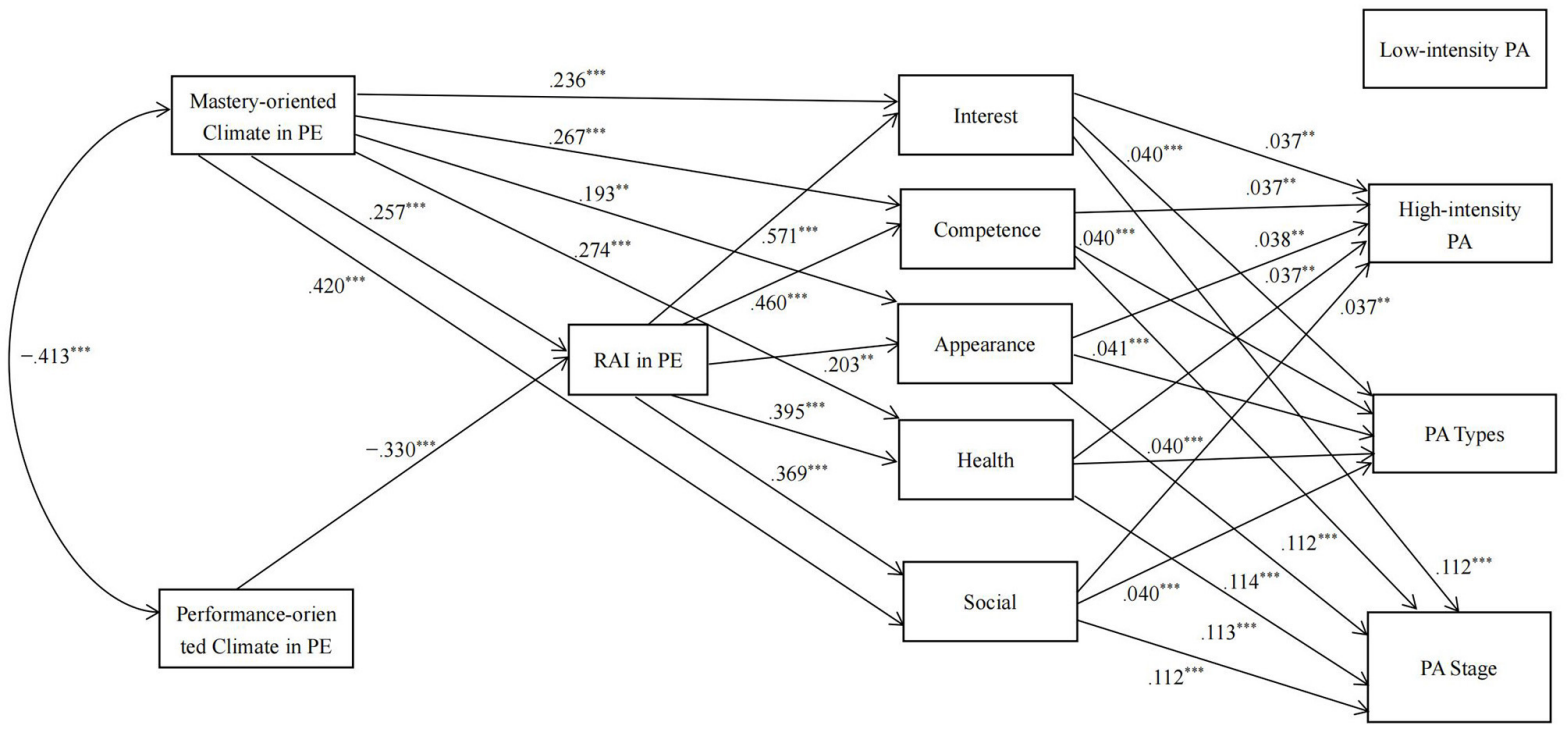
Results from the path analysis in the whole sample (controlling for gender). RAI, Relative Autonomy Index; Interest, interest motivation; Competence, competence motivation; Appearance, appearance motivation; Health, health motivation; social, social relatedness motivation; PA, physical activity. For clarity, the following parameters have been omitted from the diagram, (1) error covariances among the five types of leisure-time PA motivations, and (2) error covariances among the four PA indicators. For the sake of simplicity, the nonsignificant direct pathways are not shown in the figure. **p < 0.01, ***p < 0.001.

As shown in Table 1, a perceived performance-oriented motivational climate was unrelated to appearance motivation, the RAI in PE was unrelated to PA types, and appearance motivation was unrelated to low-intensity leisure-time PA. Therefore, we removed the direct path from the performance-oriented motivational climate to appearance motivation, the direct path from the RAI in PE to PA types, and the direct path from appearance motivation to low-intensity leisure-time PA in the path analysis. Since gender was positively related to the RAI in PE and the four types of motivation (interest, competence, social relatedness, and health) in leisure-time PA, gender was included in the path analysis as a control variable. The results of the path analysis revealed a good fit to the data [χ^2^ = 51.57, *df* = 22, *p* < 0.001; CFI = 0.987; TLI = 0.954; RMSEA = 0.065 (90% CI = 0.042, 0.088)]. The model is shown in Figure 1.

Supplementary Table 1 shows the direct effects in the path analysis. The results showed that a perceived performance-oriented motivational climate had a marginally positive effect on the PA stage (*p* < 0.1), a perceived performance-oriented motivational climate had a marginally negative effect on low-intensity PA (*p* < 0.1), and other direct effects from motivational climate to PA indicators were not significant (*ps* > 0.10).

Supplementary Table 2 shows the indirect effects in the path analysis. The results showed that the RAI in PE and the five types of motivation in leisure-time PA had significant or marginally significant chain-mediating effects on the relationship between perceived mastery- and performance-oriented motivational climates and the stage of exercise behavior change, PA types, and high-intensity PA (*p*s < 0.10). Specifically, a perceived mastery-oriented motivational climate promoted the RAI in PE, increased the five types of motivation in leisure-time PA, and led to a higher stage of exercise behavior change and engagement in more types of PA and high-intensity PA. Conversely, a perceived performance-oriented motivational climate reduced the RAI in PE, decreased the five types of motivation in leisure-time PA, and led to an earlier stage of exercise behavior change and engagement in fewer types of PA and high-intensity PA. The RAI in PE and the five types of motivation in leisure-time PA had no chain-mediating effects on the relationship between perceived mastery- and performance-oriented motivational climate and low-intensity leisure-time PA (*p*s > 0.1).

#### Chain-Mediating Effects of RAI in PE and Motivations in Leisure-Time PA in Girls

Table 2 presents the correlation coefficients for all study variables between girls and boys.

**Table 2:** Correlation coefficients between study variables in boys and girls.

	**1**	**2**	**3**	**4**	**5**	**6**	**7**	**8**	**9**	**10**	**11**	**12**
1. MO	–	−0.350**	0.465**	0.608**	0.582**	0.416**	0.602**	0.616**	0.165	0.263**	0.223*	0.287**
2. PO	−0.476**	–	−0.320**	−0.209*	−0.144	0.016	−0.167	−0.181*	−0.096	−0.083	−0.105	−0.015
3. RAI	0.364**	−0.518**	–	0.647**	0.571**	0.236**	0.526**	0.538**	0.126	0.209*	0.204*	0.443**
4. Interest	0.372**	−0.295**	0.657**	–	0.889**	0.523**	0.872**	0.733**	0.126	0.278**	0.193*	0.620**
5. Competence	0.339**	−0.214**	0.514**	0.850**	–	0.579**	0.857**	0.769**	0.157	0.290**	0.229*	0.647**
6. Appearance	0.209**	−0.108	0.315**	0.502**	0.589**	–	0.679**	0.567**	0.052	0.170	0.155	0.266**
7. Health	0.353**	−0.251**	0.500**	0.760**	0.779**	0.686**	–	0.801**	0.163	0.312**	0.253**	0.559**
8. Social	0.516**	−0.287**	0.507**	0.772**	0.777**	0.584**	0.744**	–	0.239**	0.356**	0.213*	0.561**
9. Low PA	0.132	−0.213**	0.172*	0.130	0.169*	0.103	0.116	0.152*	–	0.711**	0.162	0.230*
10. High PA	0.096	−0.142*	0.143*	0.186**	0.211**	0.062	0.099	0.142*	0.485**	–	0.152	0.330**
11. PA Type	0.059	0.022	0.005	0.189**	0.183**	0.103	0.138	0.157*	0.244**	0.341**	–	0.222*
12. PA Stage	0.192**	−0.066	0.266**	0.476**	0.475**	0.342**	0.428**	0.439**	0.120	0.218**	0.203**	–

*Coefficients in the upper triangle represent correlations among the variables for the boys. Coefficients in the lower triangle represent correlations among the variables for girls. MO, mastery-oriented climate; PO, performance-oriented climate; RAI, Relative Autonomy Index in PE; Interest, interest motivation; Competence, competence motivation; Appearance, appearance motivation; Health, health motivation; Social, social relatedness motivation; PA, physical activity; low PA, low-intensity PA time; high PA, high-intensity PA time*.

* *p < 0.05*,

** *p < 0.01*.

**Figure 2:**
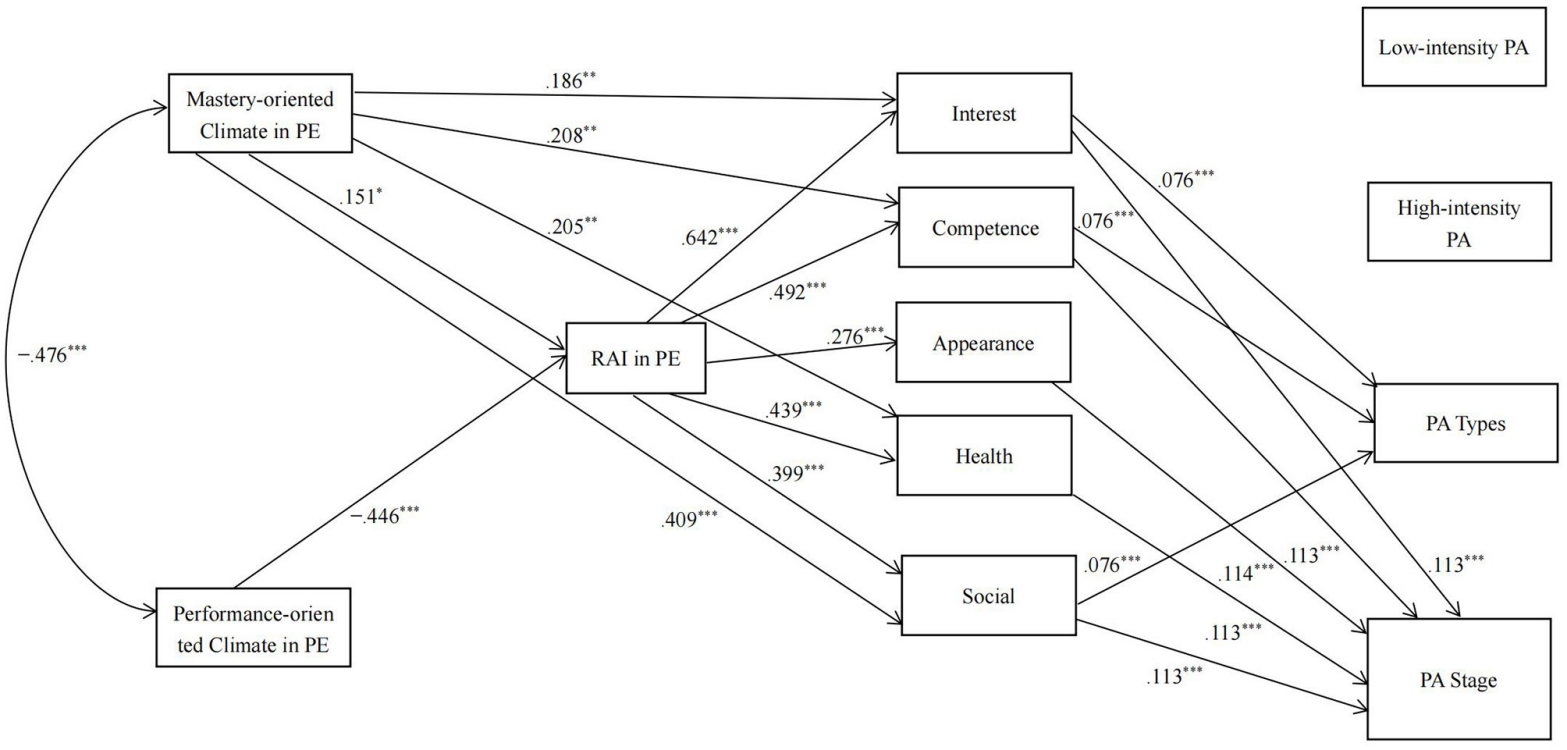
Results from the path analysis in the girls. RAI, Relative Autonomy Index; Interest, interest motivation; Competence, competence motivation; Appearance, appearance motivation; Health, health motivation; social, social relatedness motivation; PA, physical activity. For clarity, the following parameters have been omitted from the diagram, (1) error covariances among the five types of leisure-time PA motivations, and (2) error covariances among the four PA indicators. For the sake of simplicity, the nonsignificant direct pathways are not shown in the figure. *p < 0.05, **p < 0.01, ***p < 0.001.

For the girls, based on the results of the correlation analysis, we removed the direct paths from a performance-oriented motivational climate to appearance motivation, the direct path from the RAI in PE to PA types, the direct path from interest motivation to low-intensity leisure-time PA, and the direct paths from appearance motivation and health motivation to low- and high-intensity leisure-time PA and PA types. The results of the path analysis revealed a good fit to the data [χ^2^ = 16.25, *df* = 18, *p* = 0.575; CFI = 1; TLI = 1.005; RMSEA = 0 (90% CI = 0, 0.057)]. The model is shown in Figure 2.

Supplementary Table 3 shows the direct effects in the path analysis. The results showed that a perceived performance-oriented motivational climate tended to have a negative effect on low-intensity leisure-time PA (*p* < 0.1), and other direct effects from motivational climate to PA indicators were not significant (*ps* > 0.1).

Supplementary Table 4 shows the indirect effects in the path analysis. The results showed that the RAI in PE and the five types of motivation in leisure-time PA had significant or marginally significant chain-mediating effects on the relationship between perceived mastery- and performance-oriented motivational climates and the stage of exercise behavior change (*ps* < 0.1). Specifically, a perceived mastery-oriented motivational climate promoted the RAI in PE, increased the five types of motivation in leisure-time PA, and led to a higher stage of exercise behavior change. Conversely, a perceived performance-oriented motivational climate reduced the RAI in PE, decreased the five types of motivation in leisure-time PA, and led to earlier stages of exercise behavior change. In addition, the RAI in PE and three types of motivation in leisure-time PA (interest, competence, and social) had significant or marginally significant chain-mediating effects on the relationships between perceived mastery- and performance-oriented motivational climates and PA types (*p*s < 0.1). Specifically, a perceived mastery-oriented motivational climate increased the RAI in PE, increased these three types of motivation in leisure-time PA, and led to engagement in more types of PA. A perceived performance-oriented motivational climate, on the other hand, reduced the RAI in PE, decreased the three types of motivation in leisure-time PA, and led to engagement in fewer types of PA. The RAI in PE and the five types of motivation in leisure-time PA had no chain-mediating effects on the relationships between perceived mastery- and performance-oriented motivational climates and low- and high-intensity leisure-time PA (*p*s > 0.1).

#### Chain-Mediating Effects of RAI in PE and Motivations in Leisure-Time PA in Boys

**Figure 3:**
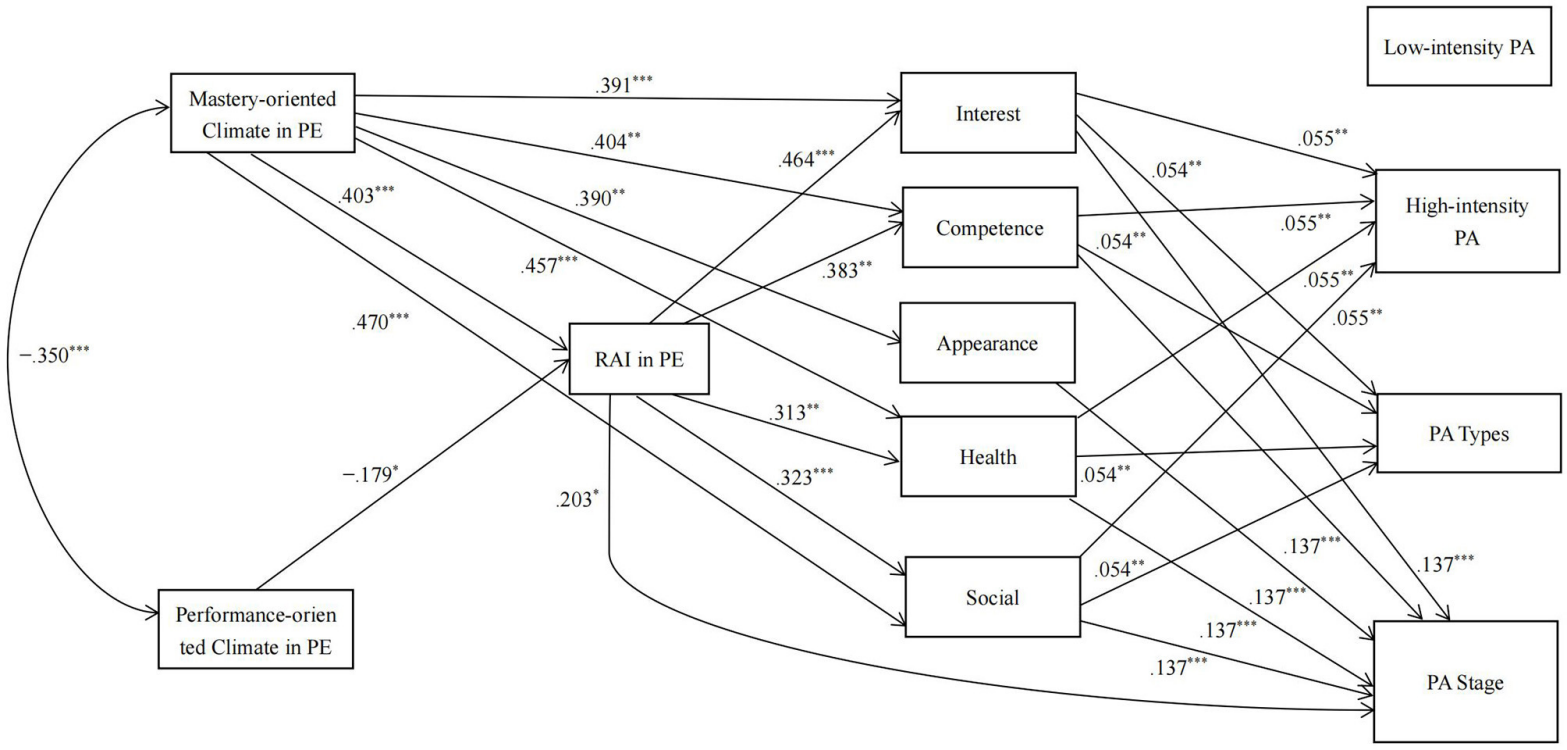
Results from the path analysis in the boys. RAI, Relative Autonomy Index; Interest, interest motivation; Competence, competence motivation; Appearance, appearance motivation; Health, health motivation; social, social relatedness motivation; PA, physical activity. For clarity, the following parameters have been omitted from the diagram: (1) error covariances among the five types of leisure-time PA motivations, and (2) error covariances among the four PA indicators. For the sake of simplicity, the nonsignificant direct pathways are not shown in the figure. *p < 0.05, **p < 0.01, ***p < 0.001.

In the group of boys, based on the results of the correlation analysis, we removed the direct paths from a performance-oriented motivational climate to competence motivation, appearance motivation, and health motivation, the direct paths from the RAI in PE, interest motivation, competence motivation, appearance motivation, and health motivation to low-intensity leisure-time PA, and the direct paths from appearance motivation to high-intensity leisure-time PA and PA types. The results of the path analysis also revealed a good fit to the data [χ^2^ = 36.22, *df* = 21, *p* = 0.021; CFI = 0.983; TLI = 0.947; RMSEA = 0.077 (90% CI = 0.03, 0.119)]. The model is shown in Figure 3.

Supplementary Table 5 shows the direct effects in the path analysis. The results suggested that a perceived mastery-oriented motivational climate tended to have a positive direct effect on high-intensity PA and a negative direct effect on the stage of exercise behavior change (*ps* < 0.1), and other direct paths from motivational climate to PA indicators were not significant (*ps* > 0.10).

Supplementary Table 6 shows the indirect effects in the path analysis. First, for the indirect effects from a perceived mastery-oriented motivational climate to PA indicators, the results showed that the RAI in PE and four types of motivation (interest, competence, social, and health) in leisure-time PA had significant or marginally significant chain-mediating effects on the relationships between a perceived mastery-oriented motivational climate and the stage of exercise behavior change, PA types, and high-intensity leisure-time PA (*p*s < 0.1). Specifically, a perceived mastery-oriented motivational climate increased the RAI in PE, increased these four types of motivation in leisure-time PA, and finally led to higher stages of exercise behavior change and engagement in more types of PA and high-intensity leisure-time PA. The RAI in PE and the five types of motivation in leisure-time PA had no chain-mediating effects on the relationship between a perceived mastery-oriented motivational climate and low-intensity leisure-time PA (*ps* > 0.1). Second, for the indirect effects of a perceived performance-oriented motivational climate on PA indicators, the results showed that the RAI in PE and four types of motivation (interest, competence, social, and health) in leisure-time PA had significant or marginally significant chain-mediating effects on the relationships between perceived performance-oriented motivational climate and the stage of exercise behavior change (*p*s < 0.1). The RAI in PE and interest motivation in leisure-time PA had marginally significant chain-mediating effects on the relationships between perceived performance-oriented motivational climate and PA types and high-intensity leisure-time PA (*p*s < 0.1). The RAI in PE and the five types of motivation in leisure-time PA had no chain-mediating effects on the relationships between a perceived performance-oriented motivational climate and low-intensity leisure-time PA (*p*s > 0.1).

In summary, autonomous motivation in PE (RAI) and the five types of motivation in leisure-time PA had chain-mediating effects on the relationships between perceived mastery- and performance-oriented motivational climates and the stage of exercise behavior change in the whole sample (controlling for gender). Similarly, a perceived mastery-oriented motivational climate tended to have a positive indirect effect on the PA types and high-intensity PA of students through chain-mediating effects of autonomous motivation in PE and the five types of motivation in leisure-time PA, while a perceived performance-oriented motivational climate had the inverse impact in the whole sample. These results corroborated the underlying mechanisms that perceived motivational climates translated into leisure-time PA. The patterns of the chain-mediating effects of autonomous motivation in PE and the five types of motivation in leisure-time PA were somewhat similar between girls and boys. Therefore, hypothesis three was confirmed.

## Discussion

Although previous studies explored the underlying mechanisms by which perceived motivational climates in PE influence leisure-time physical activity, this study contributes to the existing research by examining whether perceived motivational climates in PE could translate into the stage of exercise behavior change of students through the chain-mediating effects of autonomous motivation in PE and the five types of motivation in leisure-time PA.

Supporting the postulation of the AGT (Duda and Balaguer, 2007), this study found that, compared with a perceived performance-oriented motivational climate, a perceived mastery-oriented motivational climate could result in positive consequences; that is, autonomous motivation in PE, motivations in leisure-time PA, and actual leisure-time PA behavior. This study extended the existing research by showing the positive effect of a perceived mastery-oriented motivational climate on the stage of exercise behavior change, which provides new evidence for the effect of the motivational climate created by the PE teacher on diverse leisure-time PA indicators. In addition, the results showed that a perceived mastery-oriented climate had a positive effect on the different types of motivation in leisure-time PA, while a perceived performance-oriented climate had an inverse impact, which suggested that motivational climate may influence PA motivations beyond PE class.

Consistent with the extended TCM (Barkoukis and Hagger, 2013), we found that the perceived motivational climate in PE class could predict the stage of exercise behavior change through chain-mediating effects of autonomous motivation in PE and the five types of motivation in leisure-time PA in the whole sample (controlling gender), girls, and boys (excluding appearance motivation). Similarly, we also found that a perceived mastery-oriented motivational climate tended to have a positive indirect effect on the PA types and high-intensity PA of students through autonomous motivation in PE and the five types of motivation in leisure-time PA, while a perceived performance-oriented motivational climate had the inverse impact in the whole sample (controlling for gender), which corroborated the underlying mechanisms by which motivational climates translated into leisure-time PA. These findings suggest that we could extend the TCM by incorporating the five types of motivation (interest, competence, social relatedness, appearance, and health) that describe the specific reasons or goals for leisure-time PA.

In the whole sample (controlling gender), a perceived mastery-oriented motivational climate had a positive indirect effect on the stage of exercise behavior change by increasing autonomous motivation in PE and the five types of motivation in leisure-time PA. Additionally, we found that a perceived mastery-oriented motivational climate had a positive indirect effect on the stage of exercise behavior change by directly promoting the five types of motivation in leisure-time PA (as shown in Supplementary Table 2). These results indicate that creating a mastery-oriented motivational climate may not only stimulate the development of autonomous motivation in PE, but also directly stimulate the motivations in leisure-time PA. A mastery-oriented motivational climate offers informational feedback on the performance of students and opportunities to perform according to their capacities and individual skills, encourages them to arrange their own schedules, and encourages them to cooperate and share their own progress with their classmates, which could satisfy their need to feel competent, autonomous, and related with others, thus promoting their autonomous motivation in PE (Deci and Ryan, 1991; García-González et al., 2019). Furthermore, the autonomous motivation in PE translated into the five types of motivation in leisure-time PA, which was supportive of Vallerand (2007) model that individuals store motivational experiences in different life contexts as motivational schemas that can serve to promote or inhibit motivation toward engaging in similar contexts. Although previous studies regarded appearance and health motivation as relatively extrinsic motivations (Goguen Carpenter et al., 2017; Beauchemin et al., 2019), we found that these two types of motivation were positively related to identified motivation and not related to extrinsic motivation in PE, which supported our speculation that appearance and health motivations were closely related to identified motivation that could promote leisure-time PA (Hutmacher et al., 2020). Indeed, the five types of motivation all had positive effects on the stages of exercise behavior change of students. The findings of one cross-sectional study also revealed that these five types of motivation are positively related to the leisure-time PA of individuals (frequency of walking and distance walked; Beauchemin et al., 2019), which was corroborated in our findings. These results suggest that the five types of motivation may drive students to participate in PA regularly (Vancampfort et al., 2017). Despite this, further experimental studies are needed to confirm their effects on leisure-time PA in the future.

The perceived performance-oriented motivational climate had a negative indirect effect on the stage of exercise behavior change by decreasing autonomous motivation in PE and the five types of motivation in leisure-time PA in the whole sample (controlling gender). Compared to a mastery-oriented motivational climate, the perceived performance-oriented motivational climate is more controlling as it encourages unequal recognition, penalties for mistakes, and intra-team member rivalry. Therefore, a perceived performance-oriented motivational climate would make students feel pressured to satisfy the expectations of their teacher, incompetent, and disconnected from classmates, which would decrease autonomous motivation in PE (Deci and Ryan, 1991; García-González et al., 2019). Furthermore, the motivations in leisure-time PA were also reduced which, in turn, led to an earlier stage of exercise behavior change. However, performance-oriented motivational climate had no significant effect on the stage of exercise behavior change overall (*r* = −0.038, *p* > 0.05). Future studies could examine the predictive effect of a performance-oriented motivational climate on the stage of exercise behavior change in a longitudinal or experimental design.

The patterns of the chain-mediating effects of autonomous motivation in PE and the five types of motivation in leisure-time PA on the relationship between a mastery-oriented motivational climate and the stage of exercise behavior change were similar between girls and boys except that appearance motivation did not have a mediating effect on the relationship between a mastery-oriented motivational climate and the stage of exercise behavior change in boys. This suggested that appearance motivation played a weaker role in promoting the leisure-time PA of boys compared with other types of motivation. Unexpectedly, we found that a perceived mastery-oriented motivational climate tended to have a negative direct effect on the stage of exercise behavior change of boys in the path analysis. The reason for this unexpected result may be that boys perceived a higher level of mastery-oriented motivational climate than girls (*t* = 4.01, *p* < 0.001), while an excessive mastery-oriented motivational climate might discourage boys from participating in leisure-time PA actively when other factors remained the same because they were in a motivational climate that did not make them feel anxious to improve their athletic ability or skills to acquire praise from teachers or compare themselves with others. However, the correlation between a perceived mastery-oriented motivational climate and the stage of exercise behavior change of boys was positive (*r* = 0.287, *p* < 0.01), which indicated that a perceived mastery-oriented motivational climate had a positive effect on the stage of exercise behavior change of boys overall. We found that a performance-oriented motivational climate had a negative effect on low- and high-intensity PA among girls but not among boys, which suggests that girls might be more sensitive to a performance-oriented motivational climate than boys. Experimental studies could be conducted to test this hypothesis in the future.

Additionally, we found that girls had lower levels of autonomous motivation in PE and motivations in leisure-time PA. These results are consistent with those of Sánchez-Miguel et al. (2017), who found that boys showed higher intrinsic motivation, lower external motivation, and higher levels of PA than girls. These results suggest a tendency for girls to spend less time on PA or sports than on social activities or resting (Carson et al., 2015). Considering these findings, teaching strategies directed toward intrinsically motivating girls in PE could be beneficial. Interestingly, we also found that boys had higher levels of perceived mastery-oriented motivational climate. This may originate from varying attitudes of teachers toward gender differences. For example, if PE teachers find that boys are more active in PE, they may create a more mastery-oriented motivational climate for boys than for girls. Future studies could examine this hypothesis through observational studies in PE.

In summary, this study found that perceived motivational climates in PE translated into the stage of exercise behavior change of students through the chain-mediating effects of autonomous motivation in PE and the five or some types of motivation in leisure-time PA. These findings indicate that a mastery-oriented motivational climate should be created in PE to promote the regular engagement of students in leisure-time PA in addition to avoiding performance-oriented motivational climates.

This study had several limitations. First, there were not many PE classes of teachers involved in this study, and the sample size was not large, which may reduce the ecological validity. Second, the study was conducted using a cross-sectional design, which does not allow causal inferences. Longitudinal studies and experimental studies are needed to examine the causal relationships between perceived motivational climates and leisure-time PA participation, such as a cross-lagged design or a comparison of mastery-oriented and performance-oriented PE classes. Third, the leisure-time PA of participants was assessed using self-reported measures. Therefore, future research would benefit by using objective measures of PA participation to ensure bias is avoided.

Combining the results of this study and previous studies, it is reasonable to suggest that PE educators should create a mastery-oriented motivational climate in PE and avoid a performance-oriented motivational climate. To create a mastery-oriented motivational climate, several strategies could be used, such as providing a variety of interesting and challenging tasks and activities, providing students with meaningful choices and opportunities to participate in the decision-making process for the activities in lessons, emphasizing the efforts and cooperation of participants, reinforcing personal improvement, and allowing students to arrange their learning process (Barkoukis et al., 2008; Kokkonen et al., 2019). In addition, the results recommend avoiding intra-team member rivalry, unequal recognition, and punishment for mistakes.

## Data Availability Statement

The raw data supporting the conclusions of this article will be made available by the authors, without undue reservation.

## Ethics Statement

The studies involving human participants were reviewed and approved by Academic Ethics Committee, School of Psychology, Northeast Normal University. Written informed consent to participate in this study was provided by the participants' legal guardian/next of kin.

## Author Contributions

XW contributes to the research design, data collection and analyses, and writing. XG contributes to the research design. TY organized the database. HY and YZ provided the questionnaires they revised and gave many useful suggestions during the writing process. All authors contributed to the article and approved the submitted version.

## Funding

This study was funded by the Humanities and Social Science Planning Fund of Ministry of Education of China (Fund Number: 17YJA190003), called the development and cultivation of vocational values of middle school students.

## Conflict of Interest

The authors declare that the research was conducted in the absence of any commercial or financial relationships that could be construed as a potential conflict of interest.

## Publisher's Note

All claims expressed in this article are solely those of the authors and do not necessarily represent those of their affiliated organizations, or those of the publisher, the editors and the reviewers. Any product that may be evaluated in this article, or claim that may be made by its manufacturer, is not guaranteed or endorsed by the publisher.
