# Supplementary material for: Perceived Motivational Climate and Stages of Exercise Behavior Change: Mediating Roles of Motivation Within and Beyond Physical Education Class
Source: Front Psychol. 2021 Oct 25;12:737461. doi: 10.3389/fpsyg.2021.737461 (PMC8573023; doi:10.3389/fpsyg.2021.737461)
Supplement: Supplementary file 1 [file Table_1.docx]

**Supplemental Material**

Table S1.

*Direct effects in the path analysis with the whole sample*

|  |  |  | | **95% CI** | |  | |
| --- | --- | --- | --- | --- | --- | --- | --- |
| **Dependent variable** | **Independent variable** | **β** | **LL** | | **UL** | | ***p*** |
| RAI in PE | Mastery-oriented motivational climate | 0.257 | 0.137 | | 0.363 | | 0.000 |
|  | Performance-oriented motivational climate | −0.330 | −0.434 | | −0.229 | | 0.000 |
|  | Gender | 0.111 | 0.005 | | 0.205 | | 0.028 |
| Interest motivation | Mastery-oriented motivational climate | 0.236 | 0.118 | | 0.346 | | 0.000 |
|  | Performance-oriented motivational climate | 0.060 | −0.026 | | 0.146 | | 0.178 |
|  | RAI in PE | 0.571 | 0.465 | | 0.668 | | 0.000 |
|  | Gender | 0.185 | 0.104 | | 0.266 | | 0.000 |
| Competence motivation | Mastery-oriented motivational climate | 0.267 | 0.143 | | 0.392 | | 0.000 |
|  | Performance-oriented motivational climate | 0.081 | −0.019 | | 0.170 | | 0.090 |
|  | RAI in PE | 0.460 | 0.332 | | 0.573 | | 0.000 |
|  | Gender | 0.139 | 0.056 | | 0.222 | | 0.001 |
| Social motivation | Mastery-oriented motivational climate | 0.420 | 0.295 | | 0.527 | | 0.000 |
|  | Performance-oriented motivational climate | 0.050 | −0.024 | | 0.131 | | 0.207 |
|  | RAI in PE | 0.369 | 0.266 | | 0.475 | | 0.000 |
|  | Gender | 0.138 | 0.067 | | 0.218 | | 0.000 |
| Appearance motivation | Mastery-oriented motivational climate | 0.193 | 0.055 | | 0.332 | | 0.007 |
|  | RAI in PE | 0.203 | 0.087 | | 0.325 | | 0.001 |
| Health motivation | Mastery-oriented motivational climate | 0.274 | 0.142 | | 0.406 | | 0.000 |
|  | Performance-oriented motivational climate | 0.014 | −0.077 | | 0.101 | | 0.749 |
|  | RAI in PE | 0.395 | 0.285 | | 0.505 | | 0.000 |
|  | Gender | 0.185 | 0.105 | | 0.264 | | 0.000 |
| PA stage | Mastery-oriented motivational climate | −0.020 | −0.124 | | 0.087 | | 0.707 |
|  | Performance-oriented motivational climate | 0.091 | 0.001 | | 0.190 | | 0.061 |
|  | RAI in PE | 0.088 | −0.038 | | 0.204 | | 0.161 |
|  | Interest motivation | 0.112 | 0.083 | | 0.140 | | 0.000 |
|  | Competence motivation | 0.112 | 0.085 | | 0.137 | | 0.000 |
|  | Health motivation | 0.113 | 0.084 | | 0.140 | | 0.000 |
|  | Social motivation | 0.112 | 0.084 | | 0.139 | | 0.000 |
|  | Appearance motivation | 0.114 | 0.086 | | 0.141 | | 0.000 |
|  | Gender | 0.187 | 0.097 | | 0.276 | | 0.000 |
| Low-intensity PA | Mastery-oriented motivational climate | 0.035 | −0.099 | | 0.166 | | 0.596 |
|  | Performance-oriented motivational climate | −0.105 | −0.219 | | 0.026 | | 0.099 |
|  | RAI in PE | 0.048 | −0.083 | | 0.166 | | 0.456 |
|  | Interest motivation | 0.022 | −0.015 | | 0.058 | | 0.254 |
|  | Competence motivation | 0.022 | −0.015 | | 0.059 | | 0.254 |
|  | Health motivation | 0.022 | −0.016 | | 0.057 | | 0.250 |
|  | Social motivation | 0.022 | −0.016 | | 0.058 | | 0.256 |
|  | Gender | 0.113 | 0.000 | | 0.224 | | 0.051 |
| High-intensity PA | Mastery-oriented motivational climate | 0.049 | −0.041 | | 0.136 | | 0.277 |
|  | Performance-oriented motivational climate | −0.017 | −0.156 | | 0.108 | | 0.799 |
|  | RAI in PE | 0.036 | −0.075 | | 0.154 | | 0.536 |
|  | Interest motivation | 0.037 | 0.009 | | 0.061 | | 0.005 |
|  | Competence motivation | 0.037 | 0.009 | | 0.060 | | 0.005 |
|  | Health motivation | 0.037 | 0.009 | | 0.061 | | 0.004 |
|  | Social motivation | 0.037 | 0.010 | | 0.061 | | 0.005 |
|  | Appearance motivation | 0.038 | 0.009 | | 0.062 | | 0.005 |
|  | Gender | 0.347 | 0.255 | | 0.420 | | 0.000 |
| PA types | Mastery-oriented motivational climate | 0.018 | −0.086 | | 0.112 | | 0.714 |
|  | Performance-oriented motivational climate | 0.019 | −0.092 | | 0.128 | | 0.737 |
|  | Interest motivation | 0.040 | 0.017 | | 0.062 | | 0.000 |
|  | Competence motivation | 0.040 | 0.017 | | 0.062 | | 0.000 |
|  | Health motivation | 0.040 | 0.018 | | 0.062 | | 0.000 |
|  | Social motivation | 0.040 | 0.018 | | 0.062 | | 0.000 |
|  | Appearance motivation | 0.041 | 0.018 | | 0.062 | | 0.000 |

*Note.* RAI: Relative Autonomy Index. Gender: 0 = girl, 1 = boy.

Table S2.

*Indirect effects from perceived motivational climate to leisure-time PA indicators in the path analysis with the whole sample*

|  |  | | **95% CI** | |  | |
| --- | --- | --- | --- | --- | --- | --- |
| **Indirect effects** | **β** | **LL** | | **UL** | | ***p*** |
| ***Indirect effects from mastery-oriented motivational climate to PA stage*** |  |  | |  | |  |
| Mastery→RAI→Interest→PA stage | 0.016 | 0.009 | | 0.026 | | 0.000 |
| Mastery→RAI→Competence→PA stage | 0.013 | 0.007 | | 0.022 | | 0.001 |
| Mastery→RAI→Social→PA stage | 0.011 | 0.005 | | 0.018 | | 0.001 |
| Mastery→RAI→Health→PA stage | 0.011 | 0.005 | | 0.019 | | 0.001 |
| Mastery→RAI→Appearance→PA stage | 0.006 | 0.002 | | 0.012 | | 0.012 |
| Mastery→Interest→PA stage | 0.026 | 0.012 | | 0.043 | | 0.001 |
| Mastery→Competence→PA stage | 0.030 | 0.015 | | 0.048 | | 0.000 |
| Mastery→Social→PA stage | 0.047 | 0.029 | | 0.069 | | 0.000 |
| Mastery→Health→PA stage | 0.031 | 0.015 | | 0.051 | | 0.001 |
| Mastery→Appearance→PA stage | 0.022 | 0.006 | | 0.041 | | 0.015 |
| Mastery→RAI→PA stage | 0.023 | −0.008 | | 0.059 | | 0.192 |
| ***Indirect effects from performance-oriented motivational climate to PA stage*** |  |  | |  | |  |
| Performance→RAI→Interest→PA stage | −0.021 | −0.032 | | −0.013 | | 0.000 |
| Performance→RAI→Competence→PA stage | −0.017 | −0.027 | | −0.010 | | 0.000 |
| Performance→RAI→Social→PA stage | −0.014 | −0.021 | | −0.008 | | 0.000 |
| Performance→RAI→Health→PA stage | −0.015 | −0.023 | | −0.009 | | 0.000 |
| Performance→RAI→Appearance→PA stage | −0.008 | −0.014 | | −0.004 | | 0.005 |
| Performance→Interest→PA stage | 0.007 | −0.002 | | 0.017 | | 0.194 |
| Performance→Competence→PA stage | 0.009 | −0.002 | | 0.020 | | 0.100 |
| Performance→Social→PA stage | 0.006 | −0.003 | | 0.015 | | 0.204 |
| Performance→Health→PA stage | 0.002 | −0.008 | | 0.012 | | 0.754 |
| Performance→RAI→PA stage | −0.029 | −0.073 | | 0.011 | | 0.167 |
| ***Indirect effects from mastery-oriented motivational climate to low PA*** |  |  | |  | |  |
| Mastery→RAI→Interest→Low PA | 0.003 | −0.002 | | 0.010 | | 0.288 |
| Mastery→RAI→Competence→Low PA | 0.003 | −0.002 | | 0.008 | | 0.293 |
| Mastery→RAI→Social→Low PA | 0.002 | −0.001 | | 0.007 | | 0.292 |
| Mastery→RAI→Health→Low PA | 0.002 | −0.001 | | 0.007 | | 0.291 |
| Mastery→Interest→Low PA | 0.005 | −0.004 | | 0.015 | | 0.270 |
| Mastery→Competence→Low PA | 0.006 | −0.004 | | 0.017 | | 0.271 |
| Mastery→Social→Low PA | 0.009 | −0.007 | | 0.026 | | 0.267 |
| Mastery→Health→Low PA | 0.006 | −0.004 | | 0.018 | | 0.263 |
| Mastery→RAI→Low PA | 0.012 | −0.021 | | 0.049 | | 0.483 |
| ***Indirect effects from performance-oriented motivational climate to low PA*** |  |  | |  | |  |
| Performance→RAI→Interest→Low PA | −0.004 | −0.013 | | 0.003 | | 0.276 |
| Performance→RAI→Competence→Low PA | −0.003 | −0.011 | | 0.002 | | 0.281 |
| Performance→RAI→Social→Low PA | −0.003 | −0.008 | | 0.002 | | 0.274 |
| Performance→RAI→Health→Low PA | −0.003 | −0.009 | | 0.002 | | 0.276 |
| Performance→Interest→Low PA | 0.001 | −0.001 | | 0.007 | | 0.444 |
| Performance→Competence→Low PA | 0.002 | −0.001 | | 0.008 | | 0.399 |
| Performance→Social→Low PA | 0.001 | 0.000 | | 0.006 | | 0.465 |
| Performance→Health→Low PA | 0.000 | −0.002 | | 0.004 | | 0.806 |
| Performance→RAI→Low PA | −0.016 | −0.058 | | 0.028 | | 0.463 |
| ***Indirect effects from mastery-oriented motivational climate to high PA*** |  |  | |  | |  |
| Mastery→RAI→Interest→High PA | 0.005 | 0.002 | | 0.011 | | 0.025 |
| Mastery→RAI→Competence→High PA | 0.004 | 0.001 | | 0.009 | | 0.027 |
| Mastery→RAI→Social→High PA | 0.003 | 0.001 | | 0.008 | | 0.026 |
| Mastery→RAI→Health→High PA | 0.004 | 0.001 | | 0.008 | | 0.026 |
| Mastery→RAI→Appearance→High PA | 0.002 | 0.001 | | 0.005 | | 0.047 |
| Mastery→Interest→High PA | 0.009 | 0.002 | | 0.018 | | 0.029 |
| Mastery→Competence→High PA | 0.010 | 0.002 | | 0.020 | | 0.030 |
| Mastery→Social→High PA | 0.016 | 0.004 | | 0.028 | | 0.014 |
| Mastery→Health→High PA | 0.010 | 0.003 | | 0.021 | | 0.031 |
| Mastery→Appearance→High PA | 0.007 | 0.001 | | 0.018 | | 0.073 |
| Mastery→RAI→High PA | 0.009 | −0.020 | | 0.043 | | 0.554 |
| ***Indirect effects from performance-oriented motivational climate to high PA*** |  |  | |  | |  |
| Performance→RAI→Interest→High PA | −0.007 | −0.013 | | −0.002 | | 0.013 |
| Performance→RAI→Competence→High PA | −0.006 | −0.011 | | −0.002 | | 0.014 |
| Performance→RAI→Social→High PA | −0.004 | −0.009 | | −0.002 | | 0.012 |
| Performance→RAI→Health→High PA | −0.005 | −0.009 | | −0.002 | | 0.012 |
| Performance→RAI→Appearance→High PA | −0.003 | −0.006 | | −0.001 | | 0.032 |
| Performance→Interest→High PA | 0.002 | 0.000 | | 0.008 | | 0.256 |
| Performance→Competence→High PA | 0.003 | 0.000 | | 0.009 | | 0.170 |
| Performance→Social→High PA | 0.002 | 0.000 | | 0.006 | | 0.259 |
| Performance→Health→High PA | 0.001 | −0.003 | | 0.005 | | 0.763 |
| Performance→RAI→High PA | −0.012 | −0.053 | | 0.025 | | 0.545 |
| ***Indirect effects from*** ***mastery-oriented motivational climate to PA types*** |  |  | |  | |  |
| Mastery→RAI→Interest→PA types | 0.006 | 0.002 | | 0.011 | | 0.007 |
| Mastery→RAI→Competence→PA types | 0.005 | 0.002 | | 0.009 | | 0.008 |
| Mastery→RAI→Social→PA types | 0.004 | 0.001 | | 0.007 | | 0.008 |
| Mastery→RAI→Health→PA types | 0.004 | 0.002 | | 0.008 | | 0.009 |
| Mastery→RAI→Appearance→PA types | 0.002 | 0.001 | | 0.005 | | 0.025 |
| Mastery→Interest→PA types | 0.009 | 0.004 | | 0.018 | | 0.008 |
| Mastery→Competence→PA types | 0.011 | 0.004 | | 0.021 | | 0.009 |
| Mastery→Social→PA types | 0.017 | 0.008 | | 0.030 | | 0.003 |
| Mastery→Health→PA types | 0.011 | 0.004 | | 0.021 | | 0.009 |
| Mastery→Appearance→PA types | 0.008 | 0.002 | | 0.017 | | 0.038 |
| ***Indirect effects from*** ***performance-oriented motivational climate to PA types*** |  |  | |  | |  |
| Performance→RAI→Interest→PA types | −0.008 | −0.014 | | −0.003 | | 0.005 |
| Performance→RAI→Competence→PA types | −0.006 | −0.011 | | −0.003 | | 0.005 |
| Performance→RAI→Social→PA types | −0.005 | −0.009 | | −0.002 | | 0.005 |
| Performance→RAI→Health→PA types | −0.005 | −0.010 | | −0.002 | | 0.006 |
| Performance→RAI→Appearance→PA types | −0.003 | −0.006 | | −0.001 | | 0.022 |
| Performance→Interest→PA types | 0.002 | −0.001 | | 0.007 | | 0.220 |
| Performance→Competence→PA types | 0.003 | 0.000 | | 0.008 | | 0.135 |
| Performance→Social→PA types | 0.002 | −0.001 | | 0.006 | | 0.255 |
| Performance→Health→PA types | 0.001 | −0.003 | | 0.004 | | 0.761 |

*Note.* Mastery: mastery-oriented motivational climate; Performance: performance-oriented motivational climate; RAI: Relative Autonomy Index in PE; Interest: interest motivation; Competence: competence motivation; Appearance: appearance motivation; Health: health motivation; Social: social relatedness motivation; PA: physical activity; Low PA: low-intensity PA; High PA: high-intensity PA.

Table S3.

*Direct effects in the path analysis with the girls*

|  |  |  | | **95% CI** | |  | |
| --- | --- | --- | --- | --- | --- | --- | --- |
| **Dependent variable** | **Independent variable** | **β** | **LL** | | **UL** | | ***p*** |
| RAI in PE | Mastery-oriented motivational climate | 0.151 | 0.028 | | 0.281 | | 0.021 |
|  | Performance-oriented motivational climate | −0.446 | −0.557 | | −0.307 | | 0.000 |
| Interest motivation | Mastery-oriented motivational climate | 0.186 | 0.068 | | 0.304 | | 0.002 |
|  | Performance-oriented motivational climate | 0.099 | −0.040 | | 0.230 | | 0.160 |
|  | RAI in PE | 0.642 | 0.506 | | 0.755 | | 0.000 |
| Competence motivation | Mastery-oriented motivational climate | 0.208 | 0.070 | | 0.364 | | 0.005 |
|  | Performance-oriented motivational climate | 0.100 | −0.055 | | 0.245 | | 0.193 |
|  | RAI in PE | 0.492 | 0.352 | | 0.629 | | 0.000 |
| Appearance motivation | Mastery-oriented motivational climate | 0.109 | −0.054 | | 0.260 | | 0.170 |
|  | RAI in PE | 0.276 | 0.139 | | 0.413 | | 0.000 |
| Health motivation | Mastery-oriented motivational climate | 0.205 | 0.065 | | 0.352 | | 0.004 |
|  | Performance-oriented motivational climate | 0.024 | −0.119 | | 0.178 | | 0.746 |
|  | RAI in PE | 0.439 | 0.302 | | 0.570 | | 0.000 |
| Social motivation | Mastery-oriented motivational climate | 0.409 | 0.254 | | 0.553 | | 0.000 |
|  | Performance-oriented motivational climate | 0.076 | −0.052 | | 0.193 | | 0.211 |
|  | RAI in PE | 0.399 | 0.260 | | 0.535 | | 0.000 |
| PA stage | Mastery-oriented motivational climate | 0.021 | −0.107 | | 0.162 | | 0.757 |
|  | Performance-oriented motivational climate | 0.092 | −0.049 | | 0.229 | | 0.198 |
|  | RAI in PE | 0.032 | −0.141 | | 0.180 | | 0.689 |
|  | Interest motivation | 0.113 | 0.076 | | 0.147 | | 0.000 |
|  | Competence motivation | 0.113 | 0.078 | | 0.144 | | 0.000 |
|  | Health motivation | 0.113 | 0.078 | | 0.147 | | 0.000 |
|  | Social motivation | 0.113 | 0.079 | | 0.146 | | 0.000 |
|  | Appearance motivation | 0.114 | 0.076 | | 0.147 | | 0.000 |
| Low-intensity PA | Mastery-oriented motivational climate | −0.013 | −0.192 | | 0.136 | | 0.877 |
|  | Performance-oriented motivational climate | −0.163 | −0.344 | | 0.009 | | 0.065 |
|  | RAI in PE | 0.054 | −0.089 | | 0.190 | | 0.446 |
|  | Competence motivation | 0.056 | −0.028 | | 0.130 | | 0.167 |
|  | Social motivation | 0.056 | −0.028 | | 0.132 | | 0.169 |
| High-intensity PA | Mastery-oriented motivational climate | −0.032 | −0.189 | | 0.123 | | 0.685 |
|  | Performance-oriented motivational climate | −0.092 | −0.255 | | 0.063 | | 0.268 |
|  | RAI in PE | 0.033 | −0.111 | | 0.162 | | 0.644 |
|  | Interest motivation | 0.059 | −0.022 | | 0.118 | | 0.093 |
|  | Competence motivation | 0.059 | −0.022 | | 0.117 | | 0.090 |
|  | Social motivation | 0.059 | −0.021 | | 0.118 | | 0.092 |
| PA types | Mastery-oriented motivational climate | 0.006 | −0.129 | | 0.119 | | 0.929 |
|  | Performance-oriented motivational climate | 0.085 | −0.044 | | 0.212 | | 0.200 |
|  | Interest motivation | 0.076 | 0.033 | | 0.114 | | 0.000 |
|  | Competence motivation | 0.076 | 0.033 | | 0.113 | | 0.000 |
|  | Social motivation | 0.076 | 0.033 | | 0.115 | | 0.000 |

*Note.* RAI: Relative Autonomy Index.

Table S4.

*Indirect effects from perceived motivational climate to leisure-time PA indicators in the path analysis with the girls*

|  |  | | **95% CI** | |  | |
| --- | --- | --- | --- | --- | --- | --- |
| **Indirect effects** | **β** | **LL** | | **UL** | | ***p*** |
| ***Indirect effects from mastery-oriented motivational climate to PA stage*** |  |  | |  | |  |
| Mastery→RAI→Interest→PA stage | 0.011 | 0.002 | | 0.023 | | 0.034 |
| Mastery→RAI→Competence→PA stage | 0.008 | 0.002 | | 0.018 | | 0.042 |
| Mastery→RAI→Social→PA stage | 0.007 | 0.001 | | 0.016 | | 0.053 |
| Mastery→RAI→Health→PA stage | 0.008 | 0.002 | | 0.017 | | 0.050 |
| Mastery→RAI→Appearance→PA stage | 0.005 | 0.001 | | 0.011 | | 0.066 |
| Mastery→Interest→PA stage | 0.021 | 0.008 | | 0.038 | | 0.009 |
| Mastery→Competence→PA stage | 0.024 | 0.008 | | 0.045 | | 0.012 |
| Mastery→Social→PA stage | 0.046 | 0.026 | | 0.074 | | 0.000 |
| Mastery→Health→PA stage | 0.023 | 0.007 | | 0.043 | | 0.011 |
| Mastery→Appearance→PA stage | 0.012 | −0.005 | | 0.032 | | 0.192 |
| Mastery→RAI→PA stage | 0.005 | −0.020 | | 0.038 | | 0.727 |
| ***Indirect effects from performance-oriented motivational climate to PA stage*** |  |  | |  | |  |
| Performance→RAI→Interest→PA stage | −0.032 | −0.049 | | −0.019 | | 0.000 |
| Performance→RAI→Competence→PA stage | −0.025 | −0.042 | | −0.014 | | 0.000 |
| Performance→RAI→Social→PA stage | −0.020 | −0.034 | | −0.012 | | 0.000 |
| Performance→RAI→Health→PA stage | −0.022 | −0.038 | | −0.013 | | 0.000 |
| Performance→RAI→Appearance→PA stage | −0.014 | −0.025 | | −0.007 | | 0.003 |
| Performance→Interest→PA stage | 0.011 | −0.005 | | 0.028 | | 0.174 |
| Performance→Competence→PA stage | 0.011 | −0.006 | | 0.030 | | 0.205 |
| Performance→Social→PA stage | 0.009 | −0.005 | | 0.023 | | 0.214 |
| Performance→Health→PA stage | 0.003 | −0.013 | | 0.022 | | 0.753 |
| Performance→RAI→PA stage | −0.014 | −0.083 | | 0.067 | | 0.696 |
| ***Indirect effects from mastery-oriented motivational climate to low PA*** |  |  | |  | |  |
| Mastery→RAI→Competence→Low PA | 0.004 | −0.001 | | 0.016 | | 0.296 |
| Mastery→RAI→Social→Low PA | 0.003 | 0.000 | | 0.013 | | 0.305 |
| Mastery→Competence→Low PA | 0.012 | −0.004 | | 0.037 | | 0.239 |
| Mastery→Social→Low PA | 0.023 | −0.010 | | 0.060 | | 0.200 |
| Mastery→RAI→Low PA | 0.008 | −0.012 | | 0.040 | | 0.502 |
| ***Indirect effects from performance-oriented motivational climate to low PA*** |  |  | |  | |  |
| Performance→RAI→Competence→Low PA | −0.012 | −0.034 | | 0.005 | | 0.212 |
| Performance→RAI→Social→Low PA | −0.010 | −0.027 | | 0.004 | | 0.207 |
| Performance→Competence→Low PA | 0.006 | −0.002 | | 0.028 | | 0.408 |
| Performance→Social→Low PA | 0.004 | −0.002 | | 0.021 | | 0.414 |
| Performance→RAI→Low PA | −0.024 | −0.096 | | 0.037 | | 0.466 |
| ***Indirect effects from mastery-oriented motivational climate to high PA*** |  |  | |  | |  |
| Mastery→RAI→Interest→High PA | 0.006 | −0.001 | | 0.019 | | 0.230 |
| Mastery→RAI→Competence→High PA | 0.004 | 0.000 | | 0.014 | | 0.230 |
| Mastery→RAI→Social→High PA | 0.004 | 0.000 | | 0.013 | | 0.246 |
| Mastery→Interest→High PA | 0.011 | −0.002 | | 0.030 | | 0.187 |
| Mastery→Competence→High PA | 0.012 | −0.002 | | 0.036 | | 0.223 |
| Mastery→Social→High PA | 0.024 | −0.007 | | 0.060 | | 0.153 |
| Mastery→RAI→High PA | 0.005 | −0.017 | | 0.033 | | 0.679 |
| ***Indirect effects from performance-oriented motivational climate to high PA*** |  |  | |  | |  |
| Performance→RAI→Interest→High PA | −0.017 | −0.038 | | 0.005 | | 0.115 |
| Performance→RAI→Competence→High PA | −0.013 | −0.029 | | 0.003 | | 0.117 |
| Performance→RAI→Social→High PA | −0.010 | −0.024 | | 0.003 | | 0.113 |
| Performance→Interest→High PA | 0.006 | −0.002 | | 0.022 | | 0.333 |
| Performance→Competence→High PA | 0.006 | −0.002 | | 0.025 | | 0.364 |
| Performance→Social→High PA | 0.004 | −0.002 | | 0.018 | | 0.365 |
| Performance→RAI→High PA | −0.015 | −0.079 | | 0.049 | | 0.652 |
| ***Indirect effects from*** ***mastery-oriented motivational climate to PA types*** |  |  | |  | |  |
| Mastery→RAI→Interest→PA types | 0.007 | 0.002 | | 0.017 | | 0.057 |
| Mastery→RAI→Competence→PA types | 0.006 | 0.001 | | 0.014 | | 0.059 |
| Mastery→RAI→Social→PA types | 0.005 | 0.001 | | 0.011 | | 0.072 |
| Mastery→Interest→PA types | 0.014 | 0.004 | | 0.030 | | 0.028 |
| Mastery→Competence→PA types | 0.016 | 0.005 | | 0.036 | | 0.041 |
| Mastery→Social→PA types | 0.031 | 0.013 | | 0.054 | | 0.004 |
| ***Indirect effects from*** ***performance-oriented motivational climate to PA types*** |  |  | |  | |  |
| Performance→RAI→Interest→PA types | −0.022 | −0.038 | | −0.009 | | 0.003 |
| Performance→RAI→Competence→PA types | −0.017 | −0.030 | | −0.007 | | 0.003 |
| Performance→RAI→Social→PA types | −0.014 | −0.024 | | −0.006 | | 0.003 |
| Performance→Interest→PA types | 0.008 | −0.002 | | 0.021 | | 0.189 |
| Performance→Competence→PA types | 0.008 | −0.003 | | 0.023 | | 0.224 |
| Performance→Social→PA types | 0.006 | −0.003 | | 0.017 | | 0.240 |

*Note.* Mastery: mastery-oriented motivational climate; Performance: performance-oriented motivational climate; RAI: Relative Autonomy Index in PE; Interest: interest motivation; Competence: competence motivation; Appearance: appearance motivation; Health: health motivation; Social: social relatedness motivation; PA: physical activity; Low PA: low-intensity PA; High PA: high-intensity PA.

Table S5.

*Direct effects in the path analysis with the boys*

|  |  |  | | **95% CI** | |  | |
| --- | --- | --- | --- | --- | --- | --- | --- |
| **Dependent variable** | **Independent variable** | **β** | **LL** | | **UL** | | ***p*** |
| RAI in PE | Mastery-oriented motivational climate | 0.403 | 0.218 | | 0.569 | | 0.000 |
|  | Performance-oriented motivational climate | −0.179 | −0.339 | | −0.014 | | 0.026 |
| Interest motivation | Mastery-oriented motivational climate | 0.391 | 0.168 | | 0.594 | | 0.000 |
|  | Performance-oriented motivational climate | −0.002 | −0.069 | | 0.064 | | 0.958 |
|  | RAI in PE | 0.464 | 0.259 | | 0.657 | | 0.000 |
| Competence motivation | Mastery-oriented motivational climate | 0.404 | 0.158 | | 0.630 | | 0.001 |
|  | RAI in PE | 0.383 | 0.124 | | 0.606 | | 0.002 |
| Appearance motivation | Mastery-oriented motivational climate | 0.390 | 0.131 | | 0.626 | | 0.002 |
|  | RAI in PE | 0.055 | −0.158 | | 0.256 | | 0.607 |
| Health motivation | Mastery-oriented motivational climate | 0.457 | 0.195 | | 0.652 | | 0.000 |
|  | RAI in PE | 0.313 | 0.097 | | 0.514 | | 0.004 |
| Social motivation | Mastery-oriented motivational climate | 0.470 | 0.252 | | 0.630 | | 0.000 |
|  | Performance-oriented motivational climate | 0.011 | −0.076 | | 0.096 | | 0.791 |
|  | RAI in PE | 0.323 | 0.132 | | 0.483 | | 0.000 |
| PA stage | Mastery-oriented motivational climate | −0.161 | −0.320 | | 0.017 | | 0.057 |
|  | Performance-oriented motivational climate | 0.087 | −0.069 | | 0.252 | | 0.279 |
|  | RAI in PE | 0.203 | 0.011 | | 0.399 | | 0.041 |
|  | Interest motivation | 0.137 | 0.083 | | 0.187 | | 0.000 |
|  | Competence motivation | 0.137 | 0.086 | | 0.181 | | 0.000 |
|  | Health motivation | 0.137 | 0.087 | | 0.185 | | 0.000 |
|  | Social motivation | 0.137 | 0.088 | | 0.184 | | 0.000 |
|  | Appearance motivation | 0.137 | 0.096 | | 0.175 | | 0.000 |
| Low-intensity PA | Mastery-oriented motivational climate | 0.073 | −0.162 | | 0.318 | | 0.552 |
|  | Performance-oriented motivational climate | −0.048 | −0.223 | | 0.142 | | 0.607 |
|  | Social motivation | 0.123 | −0.110 | | 0.311 | | 0.258 |
| High-intensity PA | Mastery-oriented motivational climate | 0.124 | −0.024 | | 0.262 | | 0.094 |
|  | Performance-oriented motivational climate | 0.006 | −0.198 | | 0.206 | | 0.955 |
|  | RAI in PE | 0.021 | −0.085 | | 0.136 | | 0.708 |
|  | Interest motivation | 0.055 | 0.021 | | 0.086 | | 0.001 |
|  | Competence motivation | 0.055 | 0.021 | | 0.087 | | 0.001 |
|  | Social motivation | 0.055 | 0.021 | | 0.086 | | 0.001 |
|  | Health motivation | 0.055 | 0.022 | | 0.088 | | 0.001 |
| PA types | Mastery-oriented motivational climate | 0.061 | −0.092 | | 0.234 | | 0.462 |
|  | Performance-oriented motivational climate | −0.033 | −0.228 | | 0.153 | | 0.738 |
|  | RAI in PE | 0.038 | −0.146 | | 0.181 | | 0.642 |
|  | Interest motivation | 0.054 | 0.023 | | 0.086 | | 0.001 |
|  | Competence motivation | 0.054 | 0.022 | | 0.087 | | 0.001 |
|  | Social motivation | 0.054 | 0.022 | | 0.084 | | 0.001 |
|  | Health motivation | 0.054 | 0.023 | | 0.089 | | 0.001 |

*Note.* RAI: Relative Autonomy Index.

Table S6.

*Indirect effects from perceived motivational climate to leisure-time PA indicators in the path analysis with the* *boys*

|  |  | **95% CI** | |  |
| --- | --- | --- | --- | --- |
| **Indirect effects** | **β** | **LL** | **UL** | ***p*** |
| ***Indirect effects from mastery-oriented motivational climate to PA stage*** |  |  |  |  |
| Mastery→RAI→Interest→PA stage | 0.026 | 0.011 | 0.047 | 0.004 |
| Mastery→RAI→Competence→PA stage | 0.021 | 0.007 | 0.043 | 0.017 |
| Mastery→RAI→Social→PA stage | 0.018 | 0.007 | 0.036 | 0.010 |
| Mastery→RAI→Health→PA stage | 0.017 | 0.006 | 0.035 | 0.016 |
| Mastery→RAI→Appearance→PA stage | 0.003 | −0.009 | 0.015 | 0.620 |
| Mastery→Interest→PA stage | 0.053 | 0.020 | 0.100 | 0.011 |
| Mastery→Competence→PA stage | 0.055 | 0.018 | 0.096 | 0.005 |
| Mastery→Social→PA stage | 0.064 | 0.030 | 0.106 | 0.001 |
| Mastery→Health→PA stage | 0.062 | 0.023 | 0.111 | 0.006 |
| Mastery→Appearance→PA stage | 0.053 | 0.017 | 0.099 | 0.010 |
| Mastery→RAI→PA stage | 0.082 | 0.008 | 0.188 | 0.077 |
| ***Indirect effects from performance-oriented motivational climate to PA stage*** |  |  |  |  |
| Performance→RAI→Interest→PA stage | −0.011 | −0.025 | −0.002 | 0.047 |
| Performance→RAI→Competence→PA stage | −0.009 | −0.022 | −0.002 | 0.059 |
| Performance→RAI→Social→PA stage | −0.008 | −0.019 | −0.002 | 0.059 |
| Performance→RAI→Health→PA stage | −0.008 | −0.019 | −0.001 | 0.073 |
| Performance→RAI→Appearance→PA stage | −0.001 | −0.010 | 0.003 | 0.651 |
| Performance→Interest→PA stage | 0.000 | −0.009 | 0.009 | 0.957 |
| Performance→Social→PA stage | 0.002 | −0.011 | 0.013 | 0.794 |
| Performance→RAI→PA stage | −0.036 | −0.103 | −0.004 | 0.119 |
| ***Indirect effects from mastery-oriented motivational climate to low PA*** |  |  |  |  |
| Mastery→RAI→Social→Low PA | 0.016 | −0.008 | 0.056 | 0.326 |
| Mastery→Social→Low PA | 0.058 | −0.042 | 0.170 | 0.279 |
| ***Indirect effects from performance-oriented motivational climate to low PA*** |  |  |  |  |
| Performance→RAI→Social→Low PA | −0.007 | −0.033 | 0.003 | 0.356 |
| Performance→Social→Low PA | 0.001 | −0.009 | 0.023 | 0.845 |
| ***Indirect effects from mastery-oriented motivational climate to high PA*** |  |  |  |  |
| Mastery→RAI→Interest→High PA | 0.010 | 0.004 | 0.024 | 0.023 |
| Mastery→RAI→Competence→High PA | 0.008 | 0.003 | 0.022 | 0.056 |
| Mastery→RAI→Social→High PA | 0.007 | 0.002 | 0.017 | 0.041 |
| Mastery→RAI→Health→High PA | 0.007 | 0.002 | 0.017 | 0.050 |
| Mastery→Interest→High PA | 0.022 | 0.007 | 0.045 | 0.019 |
| Mastery→Competence→High PA | 0.022 | 0.008 | 0.045 | 0.019 |
| Mastery→Social→High PA | 0.026 | 0.010 | 0.047 | 0.006 |
| Mastery→Health→High PA | 0.025 | 0.009 | 0.052 | 0.016 |
| Mastery→RAI→High PA | 0.009 | −0.035 | 0.062 | 0.718 |
| ***Indirect effects from performance-oriented motivational climate to high PA*** |  |  |  |  |
| Performance→RAI→Interest→High PA | −0.005 | −0.012 | −0.001 | 0.096 |
| Performance→RAI→Competence→High PA | −0.004 | −0.011 | −0.001 | 0.123 |
| Performance→RAI→Social→High PA | −0.003 | −0.010 | −0.001 | 0.127 |
| Performance→RAI→Health→High PA | −0.003 | −0.009 | 0.000 | 0.129 |
| Performance→Interest→High PA | 0.000 | −0.004 | 0.004 | 0.959 |
| Performance→Social→High PA | 0.001 | −0.004 | 0.006 | 0.803 |
| Performance→RAI→High PA | −0.004 | −0.033 | 0.014 | 0.725 |
| ***Indirect effects from*** mastery-oriented motivational climate to PA types |  |  |  |  |
| Mastery→RAI→Interest→PA types | 0.010 | 0.004 | 0.022 | 0.021 |
| Mastery→RAI→Competence→PA types | 0.008 | 0.002 | 0.021 | 0.052 |
| Mastery→RAI→Social→PA types | 0.007 | 0.002 | 0.016 | 0.036 |
| Mastery→RAI→Health→PA types | 0.007 | 0.002 | 0.016 | 0.046 |
| Mastery→Interest→PA types | 0.021 | 0.007 | 0.043 | 0.018 |
| Mastery→Competence→PA types | 0.022 | 0.008 | 0.044 | 0.017 |
| Mastery→Social→PA types | 0.025 | 0.011 | 0.048 | 0.005 |
| Mastery→Health→PA types | 0.025 | 0.009 | 0.049 | 0.016 |
| ***Indirect effects from*** performance-oriented motivational climate to PA types |  |  |  |  |
| Performance→RAI→Interest→PA types | −0.005 | −0.012 | −0.001 | 0.097 |
| Performance→RAI→Competence→PA types | −0.004 | −0.012 | −0.001 | 0.124 |
| Performance→RAI→Social→PA types | −0.003 | −0.009 | −0.001 | 0.124 |
| Performance→RAI→Health→PA types | −0.003 | −0.009 | 0.000 | 0.131 |
| Performance→Interest→PA types | 0.000 | −0.004 | 0.004 | 0.959 |
| Performance→Social→PA types | 0.001 | −0.004 | 0.006 | 0.802 |

*Note.* Mastery: mastery-oriented motivational climate; Performance: performance-oriented motivational climate; RAI: Relative Autonomy Index in PE; Interest: interest motivation; Competence: competence motivation; Appearance: appearance motivation; Health: health motivation; Social: social relatedness motivation; PA: physical activity; Low PA: low-intensity PA; High PA: high-intensity PA.
